# Iron and the female athlete: a review of dietary treatment methods for improving iron status and exercise performance

**DOI:** 10.1186/s12970-015-0099-2

**Published:** 2015-10-06

**Authors:** Ieva Alaunyte, Valentina Stojceska, Andrew Plunkett

**Affiliations:** Department of Food and Tourism Management, Manchester Metropolitan University, Manchester, M14 6HR UK; Liverpool Hope University, School of Health Sciences, Liverpool, L16 9JD UK; Brunel University London, College of Engineering, Design and Physical Sciences, Uxbridge, Middlesex UB8 3PH UK; Brunel University London, Institute of Energy Futures, RCUK Centre for Sustainable Energy Use in Food Chains (CSEF), Uxbridge, Middlesex UB8 3PH UK

**Keywords:** Dietary iron, Iron-deficiency, Female athletes, Serum ferritin

## Abstract

Iron is a functional component of oxygen transport and energy production in humans and therefore is a critically important micronutrient for sport and exercise performance. Athletes, particularly female athletes participating in endurance sport, are at increased risk of compromised iron status due to heightened iron losses through menstruation and exercise-induced mechanisms associated with endurance activity. Conventionally oral iron supplementation is used in prevention or/and treatment of iron deficiency. However, this approach has been criticised because of the side effects and increased risk of iron toxicity associated with the use of supplements. Thus, more recently there has been a growing interest in using dietary modification rather than the use of supplements to improve iron status of athletes. Dietary iron treatment methods include the prescription of an iron-rich diet, or/and haem iron-based diet, dietary advice counselling and inclusion of novel iron-rich products into the daily diet. Although studies using dietary modification are still scarce, current literature suggests that dietary iron interventions can assist in maintaining iron status in female athletes, especially during intensive training and competition. Future research should focus on the most efficient method(s) of dietary modification for improvement of iron status and whether these approaches can have a favourable impact on sports and exercise performance.

## Introduction

Adequate nutrient intake is essential for achieving optimal athletic performance. Female athletes generally meet macronutrient and micronutrient requirements with the exception of iron [1, 2]. Iron is an essential micronutrient in energy production pathways and is a functional component of haemoglobin and myoglobin [3]. Female athletes are considered to be at a greater risk of compromised iron status which may lead to iron deficiency (with or without anaemia) due to negative iron balance contributed by insufficient dietary iron intake, menstruation, increased iron losses associated with haemolysis, sweating, gastrointestinal bleeding and exercise induced acute inflammation [4].

Iron deficiency treatments include oral supplements, intramuscular or intravenous injections, and dietary iron treatments such as modification of diet through dietary advice and counselling, inclusion of iron fortified products or naturally iron-rich products into the daily diet. Although conventional treatments of oral iron supplements and injections improve iron status in athletes [5], these methods often cause side effects including abdominal discomfort, constipation and nausea [6] and may present a risk of iron overload associated with unnecessary or unmonitored usage [7]. Thus dietary modification is suggested as a preferred strategy for ensuring adequate iron intake, maintenance of iron status, and as the first line of action in the prevention of iron deficiency in female athletes [8, 9].

Iron fortified products have been successfully used to increase haemoglobin, serum ferritin levels and to reduce the risk of iron deficiency in general adult population [10]. Dietary programs to improve iron status in female athletes have produced mixed results [11–13]. More recently, few studies demonstrated positive effects of incorporating naturally iron-rich products into daily diet on iron status in women of childbearing age [14] and recreational female runners [1].

This review will focus on studies investigating the effects of dietary iron treatments on iron status in female athletes. Factors affecting iron status in female athletes will be reviewed and the practical implications and challenges of improving iron status by dietary means will be discussed.

### Dietary iron

Dietary iron occurs in two forms: haem and non-haem. The haem form of iron refers to iron from animal sources, whilst non-haem iron accounts for all other types of dietary iron.

Haem iron is present within haemoglobin or myoglobin molecules and is released by proteolytic enzymes in the lumen of the stomach and the small intestine [15]. As haem iron absorption does not require binding proteins, its uptake can be as efficient as 40 % [16]. However, it should be noted, that haem iron constitutes only about 10 % of all dietary iron [15].

Non-haem iron is bound to other food components and is usually present in ferric form. In order for it to be used by the body it must be reduced to ferrous iron by either brush border membrane enzymes or dietary reducing agents and transported by the divalent metal transporter into the enterocyte [15]. Non-haem iron absorption depends on the levels of inhibitors and enhancers, hence, the availability of this form of iron varies greatly from 2 to 20 % [16]. In Western diets 50 % of total dietary iron comes from grain products [15], hence, non-haem iron accounts for the largest proportion of the iron intake for the general population.

### Iron metabolism and bioavailability

The human body does not have a direct mechanism of iron excretion, hence, regulation of iron balance is influenced by the current iron status of the individual and the total amount of iron components ingested through the diet; and is maintained by the internal homeostasis [17]. An individual with high iron stores will absorb less iron than an iron depleted person and vice versa. Furthermore, the absorption of iron from the whole diet depends on not only the total amount of iron, but also the type of iron (haem or non-haem iron) and the levels of promoters like Vitamin C, certain organic acids, meat, fish and shellfish, and inhibitors like phytates, phenolic compounds, and calcium present in the diet [18].

Researchers have demonstrated an inverse relationship between iron absorption and serum ferritin (sFer) concentration up to the level of 60 μg/L [19, 20], which suggests that absorption decreases to a level sufficient to cover daily iron losses in order to prevent further storage of iron. It was also reported that the absorption of both haem and non-haem iron was 40 % in subjects with sFer of 10 μg/L, however, at higher sFer concentration a decrease in iron absorption was observed, especially in non-haem iron [20]. This indicates that regardless of the total dietary iron intake, iron absorption is strongly dependent on overall iron status. In an iron-repleted state the absorption of iron will be reduced, whilst in the iron-depletion state, increased dietary iron absorption will occur. Furthermore, this suggests that in an iron-depletion state, non-haem dietary iron becomes an important source of absorbable iron.

### Iron deficiency

Iron deficiency progresses in three stages [21]. Firstly, iron stores in reticuloendothelial cells of the liver, spleen and bone marrow are depleted, which is observed as a fall in serum ferritin and is referred to as iron storage depletion. The second stage is represented by erythropoiesis, where transport iron is decreased and hence, iron supply to the cells is reduced. This stage is manifested as low serum iron, increased total binding capacity and a decrease in transferrin saturation. The first two stages of iron deficiency are also referred to as pre-anaemic ‘latent iron deficiency’ or ‘iron-deficient non-anaemia’. In the last stage of iron deficiency, haemoglobin synthesis falls due to insufficient iron supply, resulting in anaemia [21].

It is generally agreed that serum ferritin (sFer) is the index of iron stores in healthy subjects [15]. Plasma serum ferritin levels are strongly correlated with iron stores in the bone marrow [22], hence, low levels of serum ferritin indicate latent iron deficiency. The physiological range of serum ferritin in adult females is 15 to 200 μg/L [23]. Due to this wide inter-individual variation, the lower limit of serum ferritin for indication of latent iron deficiency in female athletes is not well established. The lower limit varies over a wide range from 12 to 35 μg/L in studies detailing iron status in population of physically active females (Table 1). A value of 12 μg/L is generally considered to indicate an iron deficiency state in females, as this state is manifested by the absence of marrow iron stores and completely depleted iron stores [24].

**Table 1:** Comparison of mean serum ferritin values and iron depletion levels in female athletes

Reference	Study design	Participants	Mean sFer (μg/L)	Cut-off value (μg/L)	Prevalence of iron stores depletion
Pate et al. [34]	Cross-sectional	Distance runners (*n* = 111)	26 ± 21	<20	50 %
Spodaryk et al. [35]	Cross-sectional	Various sports athletes (*n* = 40)	40 ± 11	<20	20 %
Sinclair and Hinton [37]	Cross-sectional	Physically active females (*n* = 72)	27 ± 28	<16	29 %
Gropper et al. [30]	Cross-sectional	Cross-country runners (*n* = 9)	38 ± 38	<12	22 %
Di Santolo et al. [38]	Cross-sectional	Various sports athletes (*n* = 70)	24 ± 17	<12	27 %
Ostojic & Ahmetovic [39]	Cross-sectional	Distance runners (*n* = 15)	27 ± 12	<12	20 %
Woolf et al. [36]	Cross-sectional	Highly active females (*n* = 28)	32 ± 28	<12	21 %
Milic et al. [40]	Cross-sectional	Various sports athletes (*n* = 359)	41 ± 22	<22	18 %
Koehler et al. [33]	Cross-sectional	Various sports athletes (*n* = 97)	35 ± 22	<35	57 %
DellaValle & Haas [41]	Cross-sectional	Rowers (*n* = 165)	NR	<20	27 %
Auersperfer et al. [44]	Longitudinal observation	Distance runners (*n* = 14)	NR	<20	50 %
Alaunyte et al. [1]	Intervention	Distance runners (*n* = 11)	30 ± 21	<12	36 %

Key: *NR* the mean value for sFer for the whole sample was not reported

### Dietary iron requirement and intakes in female athletes

The reference nutrient intake (RNI) for adult females in the UK is 14.8 mg iron per day [25] and the recommended dietary allowance (RDA) in the US is set at 18 mg iron a day [26]. Whilst additional iron is recommended for pregnant and lactating females, an increased iron allowance is not an official recommendation for female athletes. Although increased loss of several minerals, including iron, from the body during exercise has been well established, there is limited evidence of adverse effect on the body stores [27]. Some authors suggest that iron requirements for endurance female athletes, particularly distance runners, are increased by approximately 70 % [28]. If this suggestion was to be followed, a daily intake of 10 mg of iron would be added to the UK recommended value of 14.8 mg.

Some literature suggests that although female athletes generally meet their energy and micronutrient needs, they do not achieve the recommended intake of dietary iron [1, 29], whilst others report adequate intakes for this population [30]. Furthermore whether the lack of iron in the diet contributes to lower iron status in this population is opened to further debate. Poor iron intake (7–20 mg/day) and low iron stores (sFer 30 μg/L) were reported in a small sample size (*n* = 9) of female marathon runners during 11-week pre-marathon training [29] and recreational female distance runners (*n* = 11, 11 mg dietary iron/day, sFer 30 μg/L) [1]. In addition, both studies found that the intakes for most other nutrients, including daily energy, protein and Vitamin C were near recommended levels, except for iron, indicating that poor dietary iron intake might contribute to compromised iron status in this population. This is in agreement with several other cross-sectional studies investigating the influence of dietary iron sources in female runners [2, 31, 32]. In a study carried out by Nuviala et al. [32] the iron intake of 68 % of female runners (*n* = 25) was found to be below 15 mg/day. Hassapidou and Manstrantoni [2] observed similar iron intakes of 11.4 and 13.8 mg/day in middle distance runners during training and the competition season respectively, whilst Snyder et al. [31] reported dietary iron intake to be around 14 mg/day with serum ferritin ranging from 6 to 25 μg/L, indicating depleted iron stores. Interestingly, authors found that vegetarian runners had significantly (*P* < 0.05) lower serum ferritin levels (7.4 μg/L) compared to meat eating group (19.8 μg/L), which was mainly attributed to reduced iron bioavailability in the vegetarian diet (0.66 mg of absorbable iron/day *vs* 0.91 mg/day, *P* < 0.05) as both groups had a similar total iron intake [31]. Koehler et al. [33] reported a similar trend. The study indicated that low serum ferritin levels were associated with lower dietary iron density (mg per 1000 kcal) but not total dietary iron intake.

Some researchers have indicated that dietary iron intake in female athletes is similar to that of general population. In a large cross-sectional study by Pate et al. [34] daily dietary iron intake of runners was reported to be 11.0 mg/d whilst in controls it was 10.4 mg/d. In addition, dietary iron intake was associated with low serum ferritin levels in both groups. Supporting evidence was provided by a study of 40 female runners and 40 matched inactive counterparts [35], which showed that although the mean iron intake was similar in both groups (athletes 12.2 mg/d *v* controls 10.8 mg/d), iron depletion (sFer <20 μg/L) was reported in 20 % of female runners compared to 10 % in control subjects and haematological indices were significantly (*P* < 0.05) lower in athletes than sedentary women. Woolf et al. [36] similarly reported a significantly greater total dietary iron intake in highly active females when compared to inactive subjects but lower iron storage indices in physically active women, highlighting a possible negative effect of exercise on iron status.

### Association between exercise and the iron status of female athletes

The presence of iron deficiency in physically active females and endurance athletes as a result of intensive training regimens and competition has been a topic of considerable attention over the last few decades. This is due to notably high prevalence of latent iron deficiency seen in female athletes which in some cases is reported to be more than twice the level reported in their sedentary counterparts. Table 1 summarises the findings of studies detailing the prevalence of latent iron deficiency in female athletes.

The most convincing evidence highlighting the effects of exercise on iron status and the risk of iron deficiency is presented by Pate et al. [34]. Their study of 213 participants (111 habitual female runners and 65 inactive female participants) showed that an iron depletion state was significantly (*P* < 0.05) more prevalent in habitual female runners compared to the inactive counterparts. Furthermore, serum ferritin concentrations showed a significant negative correlation with running activity. Supporting evidence was provided by the following studies [1, 30, 33, 36, 37]. Researchers reported similar or even higher iron depletion levels in populations of physically active females, recreational female runners and elite female athletes. Contrary findings were reported by the subsequent studies [38, 39]. Di Santolo et al. [38] found no significant difference in the frequency of anaemia, iron-deficiency anaemia or latent iron-deficiency between physically active and inactive females. The study did however report a two to threefold lower iron status indices in non-professional female athletes compared to inactive controls. Ostojic and Ahmetovic [39] reported similar iron depletion levels in female elite athletes but only found a weak association between training duration and serum ferritin levels. A large cross-sectional study of 359 female athletes and 514 male athletes investigated haematological indices according to the predominant energy system required in difference sports [40]. The authors reported that female athletes who participate in sports which require mixed sources of energy supply (i.e. anaerobic and aerobic), such as rowing, volleyball, handball and some swimming and track and field sports, had the highest risk of iron deficiency compared to predominantly aerobic (distance running, triathlon, tennis, cross-country skiing, road cycling) or anaerobic (sprinting, swimming, alpine skiing) sports. The most plausible explanation for this observation was suggested to be the adaptive responses in muscle tissue which is subjected to a greater need for oxygen in aerobic activities. This notion is also supported by other researchers [41] who looked at the association between iron status and exercise performance in female rowers at the beginning of a training season. As expected, there were differences in exercise performance measurements, including VO_2peak_, lactate concentration and time trial between female rowers with normal iron status and those who were deficient. However this trend was only significant (*P* < 0.05) in rowers who were training ‘low’ and not those who complied with ‘hard’ training regimen. This suggests that the negative effects of intensive physical activity are more profound in less-trained athletes and/or during the adaptation period of intensive training rather than highly trained individuals.

### Dietary iron treatment methods

Studies using dietary iron treatment approach rather than pharmaceutical iron supplementation in female athletes are scarce. Dietary iron treatment methods used in literature include the prescription of an iron-rich diet [42, 43] or/and haem iron-based diet [11, 13], dietary advice counselling [12] and inclusion of novel iron-rich products into the daily diet [1]. The summary of diet modification studies is presented in Table 2.

**Table 2:** Comparison of effects of dietary iron treatment methods on iron status in female athletes

Study	Subjects	Protocol	Findings
Tsalis et al. [42]	21 male and 21 female swimmers, iron-repleted (sFer >30 μg/L, Hb > 12 g/dl) aged 12–17 years BMI 20	Study design:	- NS differences in iron status and exercise performance among 3 groups.
Supplementation and dietary randomised control trial		6-month intervention	
		A: Fe supplement (47 mg/d)	
		B: diet rich in Fe (26 mg/d)	
		C: placebo	
		Dietary assessment:	
		Records of daily food intake	
		Performance assessment:	
		Swimming tests	
Ishizaki et al. [43]	8 collegiate rhythmic gymnasts, aged 18–19 years, BMI 19.7, sFer <20 μg/L	Study design:	- SIG increased sFer and δ-ALAD activity (enzyme responsible for RBC turnover) after diet intervention.
Dietary intervention		4-weeks intervention in 2 years cohorts:	
		−1^st^ year self-selected diets	- No effect on other parameters of iron status.
		−2^nd^ year fixed diet for 4 weeks (15 mg Fe/day)	
		Dietary assessment:	
		3-day FD 3 times in 1^st^ / 2^nd^ year at baseline, 4, 8 weeks	
		Performance assessment:	
		None	
Lyle et al. [11]	60 exercising (2.3–2.5 days/week) females, previously sedentary, iron-repleted (sFer >20 μg/L, Hb > 12 g/dl) aged 18–19 years	Study design:	- Moderate aerobic exercise compromised iron status.
Supplementation and dietary randomised control trial		12-week intervention	- Meat diet was more effective in protecting Hb and iron status than were iron supplements.
		A: Fe 50 mg + low diet Fe	
		B: Fe 10 mg + low diet Fe	
		C: placebo	
		D: meat diet (18 mg Fe)	
		Dietary assessment:	
		7-day FD	
		Performance assessment:	
		Walking and treadmill tests	
Anschuetz et al. [12]	12 male and 5 female collegiate middle distance runners and 6 male and 2 female non-running controls, aged 18–24 years, sFer <30 μg/L	Study design:	- sFer SIG greater in MHIA than LMIA group.
Dietary advice counselling intervention		4-week dietary advice counselling intervention in LMIA group only	- SIG correlations between absorbable dietary Fe and sFer, sFe, TIBC and Hb.
		LMIA: low/medium Fe availability diet	- No significant difference in iron status indices between LMIA and MHIA groups post-intervention
		MHIA: medium/high Fe availability diet	
		C: control	
		Dietary assessment:	
		3-day FD	
		Performance assessment:	
		None	
Burke et al. [13]	14 male and 14 female collegiate cross country runners, aged 18–24 years, sFer >60 μg/L	Study design:	- NS improvements in haematocrit and TIBC in intervention group females.
Dietary randomised control trial		8-week intervention	- No differences between the groups in other parameters of iron status.
		IG: 9 oz lean beef/week	- No difference in VO_2max_ changes between the groups.
		C: control group	
		Dietary assessment:	
		3-day FD	
		Performance assessment:	
		Treadmill tests	
Alaunyte et al. [1]	11 female runners, recruited from running clubs, aged 20–44 years, BMI 23, sFer >30 μg/L	Study design:	- NS improvements in iron
Dietary intervention		6-week intervention (18.5 mg Fe/d)	- SIG correlations between ↑ dietary iron and ↑ sFer (*r* = 0.8, *P* < 0.05).
		Dietary assessment:	
		Multiple 24-h recalls	
		Performance assessment:	
		None	

*Abbreviations: PA* physical activity, *SIG* significant, *NS* non significant, *IG* intervention group, *C* control group, *FD* food diary, *FFQ* food frequency questionnaire, *Hb* haemoglobin, *Fe* iron, *sFe* serum iron, *sFer* serum ferritin, *TIBC* total iron binding capacity, *RBC* red blood cells, *δ-ALAD* Delta-aminolevulinic acid dehydratase

A longitudinal study showed no differences in iron status or swimming performance between an iron-rich diet and free choice diet in swimmers during a 6-month period [42]. It should be noted that the study recruited iron-repleted participants (Hb > 14 g/dL, sFer >30 μg/L), hence, the failure of high dietary iron intake to change iron status may be attributed to the homeostatic mechanisms and may explain the lack of effect. Other dietary intervention studies have demonstrated more positive effects on iron status in female athletes. A four week iron-rich diet, which provided 18.2 mg of iron daily, resulted in significantly higher serum ferritin concentrations in rhythmic gymnasts [43]. The study however did not assess their performance during the trial and hence, no conclusions can be drawn as to any possible effects on exercise performance. A study by Lyle et al. [11] reported that a diet rich in meat protein, providing 11.8 mg of daily dietary iron, was more effective in protecting iron status than were supplements (50 mg ferrous sulphate/day), during a 12-week aerobic training trial, in previously sedentary women. Furthermore, the dietary intervention group showed the greatest improvements in exercise performance highlighting the possibility that dietary iron may play a role during exercise adaptation.

A dietary counselling intervention study conducted by Anschuetz et al. [12] failed to demonstrate any significant improvements in the iron status of female distance runners after a 4-week dietary education intervention. Nevertheless, the authors suggested that the diet composition, in particular the presence of enhancers of non-haem iron absorption, has a significant influence on iron absorption in this population. The study findings revealed that dietary absorbable iron was significantly correlated to serum ferritin (*r* = 0.9, *P* < 0.05) in female mid-distance runners, highlighting the importance of the levels of absorbable iron in the diet [12]. The importance of iron bioavailability in dietary intervention was also studied by other researchers, who investigated the effects of the addition of lean beef to the diets of distance runners, during a competitive season [13]. The authors reported no significant differences between the intervention and control groups in iron parameters during a period of 8 weeks. In fact, some of the iron status indices were found to have decreased in both groups, which may have been due to their intensive training regimens. Nevertheless, gender evaluation revealed that female runners, as a result of the dietary intervention, experienced a near significant change in haematocrit levels (*P* = 0.055). The most recent study investigating iron status in female athletes used dietary intervention, incorporating a novel and naturally iron-rich Teff bread into the daily diets of female runners [1]. A six week dietary intervention, which provided 18.5 mg of iron per day, showed no significant improvement in iron status. The authors did however report significant correlations between the increase in dietary iron intake and changes in serum ferritin concentration.

### Practical implications and Conclusion

Conflicting evidence still exists as to whether the higher prevalence of iron deficiency in female athletes is a result of the intensive physical activity or/and inadequate iron intake, and more importantly whether mild iron deficiency impairs sport and exercise performance. It appears that depleted iron stores can be the result of intensive training, especially during the adaptation to exercise/training period or when undertaken by less well trained individuals. In addition, inadequate dietary iron intake and in particular the level of absorbable and bioavailable iron in the diet, may further contribute to depletion of stored iron.

Practical considerations for the maintenance or improvement of iron in female athletes should incorporate dietary modifications centred on healthy eating practices with particular focus on increasing total dietary iron, especially haem-iron intake, and improving iron bioavailability by altering meal composition. For instance, iron-rich foods can be consumed with fruit and vegetables, enhancing iron absorption due to the presence of higher levels of Vitamin C. Similarly, the intake of iron absorption inhibitors such as tannins in tea or coffee or calcium in milk, can be decreased or at least avoided in the same meal.

To conclude, the majority of research studies support the hypothesis of the beneficial effect of dietary iron interventions on the balance of iron in iron-depleted female athletes. However, the direct impact on exercise performance among female athletes is unclear. Nevertheless, there seems to be evidence that dietary iron interventions may assist in maintaining iron status in female athletes, especially during intensive training and competition regimens.

### Availability of supporting data

All the data from this research is available on your request.
